# Effects of Partial Replacement of Nitrogen Fertilizer with Organic Fertilizer on Rice Growth, Nitrogen Utilization Efficiency and Soil Properties in the Yangtze River Basin

**DOI:** 10.3390/life13030624

**Published:** 2023-02-23

**Authors:** Jiabao Wang, Xiangming Zhang, Manman Yuan, Gang Wu, Yixiang Sun

**Affiliations:** 1 Institute of Soil and Fertilizer, Anhui Academy of Agricultural Sciences, Hefei 230031, China; 2 Key Laboratory of Nutrient Cycling, Resources and Environment of Anhui, Hefei 230031, China

**Keywords:** organic fertilizer substitution, nitrogen efficiency, nutrient absorption, rice, soil properties

## Abstract

Cake fertilizer and dairy manure were used as experimental materials to carry out a 9-year (2012–2020) field experiment in the main rice production areas in the Yangtze River basin. Different fertilization modes were used (no fertilization, CK; chemical fertilizer application alone, HY; reduced fertilization with chemical fertilizer application, RF; cake fertilizer replacement of nitrogen fertilizer, CFR; and dairy manure replacement of nitrogen fertilizer, DMR). Changes in the total rice yield, yield components, absorption of nitrogen, soil pH, organic matter, total nitrogen, and soil bulk density under different fertilization treatments were analyzed. The results show that organic fertilizer replacement leads to a stable and high rice yield. The 9-year average rice yields of the CFR and DMR treatments were 60.0% and 61.5% higher than that of CK. The nitrogen uptake of the CFR and DMR treatments was also higher than that of the other treatments. The nitrogen recovery efficiency in the current season could be increased by 16.37–22.89%, and after 9 years of testing, the soil total nitrogen contents of CFR and DMR increased by 0.23–0.85 g·kg^−1^ compared to the other treatments. The available P and K contents of DMR increased by 30.17 mg·kg^−1^ and 22.02 mg·kg^−1^ compared with HY, respectively. The soil bulk density was reduced by 0.08 g·cm^−3^. Generally, the effects of dairy manure replacement were better than those of cake fertilizer. This is an important method that can be used to fertilize the soil and foster sustainable soil utilization in the rice-growing area of the Yangtze River Basin, as a long-term partial replacement for chemical nitrogen fertilizer.

## 1. Introduction

The Yangtze River Basin is China’s largest rice-producing area, and it accounts for 39.1% and 42.0% of the country’s total planting area and total rice yield, respectively [1]. Achieving a high rice cultivation yield and efficiency in this region is, therefore, crucial for ensuring food security in China. The application of chemical fertilizers can significantly increase the crop yield [2]. However, with a continuous increase in chemical fertilizer application, its utilization efficiency will decrease, and the effect of fertilization on the crop yield increase will gradually decline. The increased fertilizer application will induce a loss of nitrogen and increase the effects of greenhouse gas emissions. Long-term high investment not only increases production costs and wastes resources but also has large negative impacts on the environment [3,4,5]. The TN emissions from water pollution in the seven major river basins of China, including the Yangtze River Basin, accounted for 89.5% of the total TN emissions in 2017. The TN emissions from agricultural water sources amounted to 141.49 million tons, of which the crop, livestock, and poultry farming industries accounted for more than 90% [6]. Livestock manure and cake fertilizer account for 67% of organic fertilizer resources in China, being equivalent to more than 50 million tons of N + P_2_O_5_ + K_2_O nutrients; however, only around 50% of organic fertilizer resources are reincorporated into the field [7]. Abandoned organic fertilizer resources not only cause serious environmental problems but are also a waste of valuable fertilizer resources.

The combined application of organic and chemical fertilizers is an effective technology implemented to improve soil physical and chemical properties, enhance the soil water and fertilizer conservation capacity, and promote a high and stable rice yield [8]. Proper fertilizer replacement with organic fertilizer can aid in the absorption of organic waste and improve the ecological environment, reduce the fertilizer input, and promote nutrient cycling and green development in agriculture [9]. The results of 34 consecutive years of a positioning experiment in the Jiangxi double-harvest rice-growing area of China showed that the total yield, yield stability, and soil physiochemical properties of the early- and late-harvested rice increased after the application of a regime that used organic fertilizer to replace 70% of the chemical fertilizer [10]. Zhang et al. [11] conducted research for 10 consecutive years on organic replacement in a facility-implemented vegetable planting system and found that with a 50% proportion of organic fertilizer replacement, soil P conversion and availability were significantly promoted, while the crop yield increased by 6.9% to 18.1%. A study based on four long-term positioning experiments in China showed that the combined application of organic fertilizer increased the nitrogen utilization rate by 14% [12]. However, although combining chemical and organic fertilizers can effectively reduce the soil bulk density and increase the soil organic matter content, it may have almost no impact on the crop yield [13,14]. It can be seen that the effects of organic fertilizer on crop growth after the partial replacement of chemical fertilizers remain controversial.

Previous studies on organic fertilizer replacement have focused on a single plant- or animal-sourced organic fertilizer [10,15], and few studies have simultaneously considered different sources of organic fertilizers [16]. In this study, we used cake fertilizer and dairy manure, two different sources of organic fertilizer commonly used on farmland, as experimental materials and carried out a 9-year field positioning experiment in the rice-planting area of the Yangtze River basin. We expected this study to prove that the long-term application of organic fertilizer from two different sources can contribute to rice growth, nutrient absorption and utilization, and the improvement of soil properties. This research is conducive to efforts aiming to clarify a fertilization strategy that is suitable for maintaining or improving rice yields and reducing fertilizer input in the Yangtze River basin.

## 2. Materials and Methods

### 2.1. Overview of the Experimental Area

The experimental area is located in the middle and lower reaches of the Yangtze River in Jianhua Village, Zhongshan Town, Hefei City, Anhui Province (N31.6584, E117.7866). This region falls within the north subtropical monsoon climate zone, with an altitude of 15 m and an annual average rainfall of 1160 mm, which is mainly concentrated during the May–June plum rain season, as well as an annual average temperature of 16.2 °C. The experimental area is subject to alternate plantings of rice and wheat, with two harvests per year. The soil type is gley rice soil [17]. The soil surface physiochemical properties before the experiment were as follows: pH 6.93 (1:5 soil/water), total N 1.52 g∙kg^−1^, available P 13.10 mg∙kg^−1^, available K 128.69 mg∙kg^−1^, and organic matter 28.74 g∙kg^−1^.

### 2.2. Experimental Design, Treatments, and Cropping Practices

The experiment was conducted from June 2012 to October 2020, and five treatments were implemented, namely no fertilizer (CK), a high-yield treatment with chemical fertilizer (N 270 kg·hm^−2^ application; HY), reduced fertilization treatment with chemical fertilizer (N 210 kg·hm^−2^ application; RF), cake fertilizer replacement treatment with chemical fertilizer (N 210 kg·hm^−2^, with cake fertilizer as a replacement for chemical fertilizer N 60 kg·hm^−2^; CFR), and dairy manure replacement treatment with chemical fertilizer (N 210 kg·hm^−2^, with dairy manure fertilizer as a replacement for chemical fertilizer N 60 kg·hm^−2^; DMR). A randomized block design was used, with three replicates. The area of each plot was 36 m^2^ (4 m width × 9 m length), and the plots were arranged randomly. The ridge between the plots was covered with waterproof cloth to prevent water run-off. All treatments contained the same amount of 60 kg P_2_O_5_ hm^−2^ and 90 kg K_2_O hm^−2^ fertilizer, except for CK. The nitrogen fertilizer for each treatment was prepared as a base fertilizer, with a tiller fertilizer:panicle fertilizer ratio of 5:3:2, and P and K fertilizers were applied once with organic fertilizer as a base fertilizer.

Rice varieties with a large planting area in the current season were selected for use as test rice, including “Liangyou 1128” (2012–2015), “Y Liangyou 900” (2016–2017), and “Hui Liangyou 280” (2018–2020). The test rice was sown on 15–25 May according to the instructions for the selected rice varieties, as well as the meteorological conditions of the experimental area in the specific year, and then manually transplanted on 20–30 June. The harvest period generally occurred between middle to late October. The seedlings were planted at a rate of 225,000 holes hm^−2^, with a row interval of 33.3 cm and a planting interval of 13.3 cm. The chemical fertilizers used in the experiment were large-grained urea (46% N), common calcium superphosphate (12% P_2_O_5_), and potassium chloride (60% K_2_O). The organic fertilizers used were ranched dairy manure purchased from Anhui Lu’an Yiniu Biotechnology Co., Ltd. (pH 6.22, EC 6.13 ms·cm^−1^, water content 8.89%; 0.37% N, 0.22% P_2_O_5_, and 0.28% K_2_O) and rapeseed cake, purchased from a local rapeseed oil mill (pH 9.19, EC 8.93 ms·cm^−1^, water content 15.94%; 5.20% N, 1.96% P_2_O_5_, and 1.34% K_2_O). An adequate water layer (3–5 cm) was maintained for approximately 10 days after transplanting and within 7 days after fertilization, during both the booting and filling stages. Alternate wet and dry irrigation methods were used from the tillering to the pre-booting stages. The water was drained in the critical leaf stage of effective tillering and left to dry naturally before harvest. All disease, pest, and grass control measures, as well as other field management measures, were the same in all the treatments, except fertilization.

### 2.3. Data Collection

#### 2.3.1. Grain Yield and Yield Components

The rice yield for each plot was measured by manual harvesting and threshing. The grains were sun-dried to adjust the moisture content to 13.5%. In the mature stage, 10 holes of rice plants from each plot were sampled at random and averaged to estimate the effective panicle number (PT), number of grains per panicle (SP), seed setting rate (PFG), and 1000-grain weight (TGW).

The coefficient of variation (*CV*) was used to describe the average rice yield stability in the different experimental years. Specifically, its value is inversely proportional to the yield stability [18].

$$CV=\frac{S}{x}$$

The Sustainable Yield Index (*SYI*) reflects rice yield sustainability. The higher the value, the better the rice yield sustainability [19].

$$SYI=\frac{\left(x-\left.S\right)\right.}{X_{\max}}$$

*S* = Standard deviation of the average rice yield, kg·hm^−2^;
*X* = Average rice yield, kg·hm^−2^;
*X*max = Maximum rice yield during the experiment, kg·hm^−2^.

#### 2.3.2. Plant Dry Matter Accumulation in Different Growth Stages

In 2020, 5 holes of rice plants (excluding roots) were selected from each plot to estimate the total aboveground biomass. After drying in an oven at 80 ℃, the straw was weighed in the jointing and heading stages, while the grain and straw were weighed in the mature stage.

#### 2.3.3. Nitrogen Use Efficiency

We conducted a 9-year experiment, partly replacing chemical fertilizer with organic fertilizer, but our research only discusses the nitrogen uptake and nitrogen fertilizer utilization efficiency of organic fertilizer as a partial replacement for chemical fertilizer in the 9th year. To determine the nutrient concentration of the grain and straw, plant samples were boiled with H_2_SO_4_-H_2_O_2_. The N, P, and K concentrations in the resultant liquid were tested using the Kjeldahl method for N, while vanadium–molybdenum–yellow colorimetry was used for P, and a flame photometer was used for K [20]. Based on this, we calculated the fertilizer nutrient uptake.

We calculated the fertilizer nutrient use efficiency as follows:

$${RE}_{N}=\frac{N-N0}{F}\times 100\%$$

$${AE}_{N}=\frac{Y-Y0}{F}$$

$${PFP}_{N}=\frac{Y}{F}$$

*RE_N_* = Nitrogen recovery efficiency in the current season, %;
*N* = Nitrogen accumulation with applied nitrogen, kg·hm^−2^;
*N*0 = Nitrogen accumulation in the control treatment, kg·hm^−2^;
*F* = Amount of fertilizer, kg·hm^−2^;
*AE_N_* = Agronomic nitrogen use efficiency, kg·kg^−1^;
*Y* = Crop yield with applied nitrogen, kg·hm^−2^;
*Y*0 = Crop yield in the control treatment, kg·hm^−2^;
*PFP_N_* = Partial productivity of nitrogen fertilizer, kg·kg^−1^.

#### 2.3.4. Soil Sample Collection and Determination of Physiochemical Properties

A 5-point sampling method was used to collect the surface soil (0–20 cm) before the start (May 2012) and after the end (October 2019) of the 9-year experiment. Samples were air-dried naturally in the laboratory and passed through 20- and 100-mesh sieves for later use. The Walkley–Black method was used to determine the number of organic materials [21]. The soil total N was determined by the semi-trace Kjeldahl method [22]. The soil available P was extracted using 0.5 mol·L^−1^ NaHCO_3_ and determined by the vanadium molybdate blue colorimetric method [23]. The soil available K was extracted using NH_4_OAc and determined by flame spectrophotometry [24]. The soil pH in the soil extract was estimated (soil–water ratio of 1:2.5) [25]. At the end of the experiment (October 2020), three points in each plot were selected after rice harvesting, and the soil bulk density was measured using the ring knife method [26].

### 2.4. Statistical Analysis

The experimental data were processed with EXCEL 2010. General Linear Models in SPSS22.0 were used for the statistical analysis. A two-factor ANOVA model, using an interaction, was used to evaluate the year (Y), treatment (T), and year × treatment (Y × T). Finally, the LSD method was used for significance testing at P = 0.05.

## 3. Results

### 3.1. Effects of Organic Fertilizer as a Partial Replacement for Chemical Fertilizer on Rice Yield Components and Grain Production

The effects of fertilization on all the rice yield components were significantly different, while the number of years also affected the rice yield components, except for the number of grains per panicle, which differed significantly. The interaction between the two had no significant effect on the yield components. Between 2016 and 2020, the numbers of grains per panicle for CFR and DMR, treated with organic fertilizer replacement, were higher compared to that of HY, and the averages for year 5 were 2.1% and 2.8% higher compared to that of HY, respectively, while the 1000-grain weight of the rice tended to increase after applying organic fertilizer as a partial replacement for chemical fertilizer. This indicated that when organic fertilizer partially replaced chemical fertilizer for a certain number of experimental years, the rice yield was improved, with an increased number of grains per panicle and 1000-grain weight. Different fertilization treatments had little effect on the rice seed setting rate (Table 1).

The year and fertilization significantly influenced the rice yield, with their interaction also being highly significant (Table 2). Fertilization significantly increased the rice yield. In year nine, the yield of rice treated with fertilization was significantly higher compared to that of CK without fertilization. The yields of HY, RF, CFR, and DMR increased by 56.9%, 46.2%, 60.0%, and 61.5% compared to CK, respectively. In the first three years of the experiment (2012–2014), when the rice was treated with high nitrogen application, HY was the highest, but from year 4 onwards (2015), the values for the DMR treatments were all higher compared to that of HY, while CFR was only slightly lower compared to HY in 2020. The average rice yields of CFR and DMR in year 9 were 2.0% and 2.9% higher compared to that of HY, respectively. In addition, compared with chemical fertilizer alone, the rice yield CV for the organic fertilizer replacement decreased by 0.01–0.05, while the SYI increased by 0.006–0.094. Organic fertilizer replacement did not affect the rice yield during the first few years of the experiment. However, as the number of experimental years increased, the rice yield tended to stabilize and even slightly increased.

### 3.2. Effects of Organic Fertilizer as a Partial Replacement for Chemical Fertilizer on the Rice Straw Yield and Total Biomass Production

Fertilization can significantly increase the rice straw yield, total biomass, and the rice harvest index. In the early stage of the experiment, the straw yield of the HY treatment with chemical fertilizer alone was lower than that of the CFR and DMR treatments with organic substitution (except in 2012). From 2016 to 2020, the straw yield of HY was higher than that of CFR and DMR, but the total biomass of each treatment showed the opposite pattern. The harvest index of HY showed a trend of first increasing and then decreasing with the increase in the number of test years, but the harvest index of CFR and DMR was relatively stable and remained high. This shows that long-term organic fertilizer can promote the transfer of nutrients to grains when replacing chemical fertilizer, thus improving the crop harvest index. The above indices of RF for the treatment with reduced fertilizer application were lower in the test period (Table 3).

### 3.3. Effects of Organic Fertilizer as a Partial Replacement for Chemical Fertilizer on the Nutrient Concentrations and Nutrient Uptake in Plants

**Figure 1:**
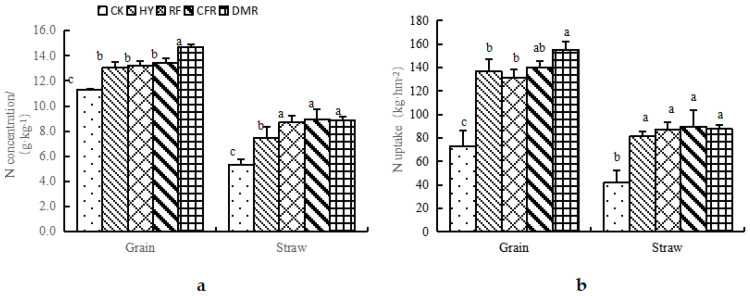
N concentration (**a**) and uptake (**b**) in rice grain and straw aboveground absorption with different fertilization treatments (2020). Note: Different lowercase number indicate significant difference between treatment, *p* < 0.05.

It can be seen from Figure 1a that the nitrogen concentration of the rice grain under different fertilization treatments is between 11.33 and 14.69 g kg^−1^, and fertilization could significantly increase the nitrogen concentration of the rice grain and straw. The grain nitrogen concentrations of the treatments follow the order of DMR > CFR > RF > HY > CK. The grain nitrogen concentration of DMR is significantly different from that of the other treatments, which indicates that organic fertilizer, as a partial replacement for chemical fertilizer, can increase the grain nitrogen concentration of rice, and that of DMR is significantly increased. The nitrogen concentration of the CFR straw is the highest, and there is a significant difference between CK and RF. However, there was no significant difference between the nitrogen concentrations of the CFR and DMR straw with the HY treatment. Likewise, there was no significant difference in straw nitrogen concentration between DMR, CFR, and HY.

Fertilization can significantly increase the nitrogen uptake of grain and straw (Figure 1b). Organic fertilizer, as a partial replacement for chemical fertilizer, can increase the nitrogen uptake of grain and straw at the same time, but compared with the application of chemical fertilizer alone, the increase in the nitrogen uptake of straw is less than that of grain. The grain nitrogen uptake of DMR was the highest, reaching 155.39 kg hm^−2^, which is significantly higher than that of HY and RF.

### 3.4. Effects of Organic Fertilizer as a Partial Replacement for Chemical Fertilizer on the Nutrient Harvest Index and Fertilizer Use Efficiency of Rice

There was a significant difference in the nitrogen fertilizer use efficiency between the different fertilization treatments. The RE_N_ of CFR and DMR, with organic fertilizer as a partial replacement for chemical fertilizer, was 16.37% and 22.89% higher compared to that of HY with chemical fertilizer application alone, respectively, and the results were significantly different. However, the difference was not significant compared with RF. The N agricultural utilization rate of CFR and DMR was 2.21–4.77 kg/kg higher than that of HY and RF, but this was insignificant. The partial N productivities of CFR and DMR were 10.95 kg/kg and 11.59 kg/kg higher than that of HY, respectively, and were significantly different. DMR had the highest nitrogen harvest index (63.89%), but no significant difference between treatments was observed (Table 4).

### 3.5. Effects of Organic Fertilizer as a Partial Replacement for Chemical Fertilizer on Soil Chemical and Physical Properties

The 9-year continuous rice positioning experiment showed that the pH values of the different fertilization treatments were significantly higher than the initial value of the soil (pH 6.93), indicating that the long-term application of organic fertilizer to neutral soil does not cause soil acidification. Fertilization can increase the content of soil organic matter. Compared with the initial value (28.74 g·kg^−1^), different treatments could increase the content of soil organic matter in a range from 2.79 to 10.69 g·kg^−1^. The effect of DMR on the soil organic matter was significantly higher than that of CFR. The 9-year positioning experiment showed that, except for CK, the total N content of all the other treatments was higher than the initial value (1.52 g·kg^−1^), and DMR had the largest increase (48.7%). There were also significant differences in soil available P between the different treatments. The available P content for DMR with organic fertilizer as a partial replacement for chemical fertilizer was significantly higher compared to that of the other treatments, while the values of CK and RF were both lower than the initial value (13.1 mg·kg^−1^). Although the value for CK was slightly lower than the initial value (128.69), the other treatments increased the available P to varying degrees. Moreover, the soil available K content of DMR was significantly higher compared to that of the other treatments. This is mainly because the straw returning mode is adopted in the test area, and the potassium concentration in the straw is high, so that it is mineralized after being applied into the soil. Organic fertilizer as a partial replacement for chemical fertilizer reduced the soil bulk density to varying degrees. The soil bulk density of DMR was lower compared to that of CFR and significantly lower compared to that of CK and RF, while that of CFR was significantly lower compared to CK (Table 5).

## 4. Discussion

### 4.1. Rice Yield Components and Grain Production

The effective panicle number, number of grains per panicle, seed setting rate, and 1000-grain weight determine the rice yield formed in different growth stages [27]. In this study, the partial substitution of chemical fertilizer with organic fertilizer was mainly intended to improve the rice yield by increasing the number of grains per panicle and the weight per 1000 grains. After the 5th year of the experiment (2016–2020), the number of grains per panicle and the 1000-grain weight increased under the treatment with organic fertilizer partly replacing chemical fertilizer. Previous studies have shown that after partly replacing chemical fertilizer with organic fertilizer, the number of rice panicles and grains per panicle increased, but there was little impact on 1000-grain weight [28]. This may be caused by different rice varieties and planting environments.

The partial replacement of chemical fertilizer nutrients with organic fertilizers can better mitigate the problems associated with nutrient loss and crop growth requirements, thereby achieving a higher yield [29]. In year 3 of our experiment, the rice yield of the HY treatment with chemical fertilizer application alone was higher compared to that of CFR and DMR applied similarly. This was mainly because during the early stage of the experiment, fertilizer nutrients could be released quickly. In contrast, the organic fertilizer nutrients required a slower process of leaching, enrichment, and mineralization release [30], thus leading to lower rice yields compared to chemical fertilizer alone during the early stage of the experiment. However, as the number of experimental years increased, the advantage of long-term organic fertilizer nutrient release gradually became apparent. From year 4 onward and in year 9, the rice yield of CFR was slightly lower compared to that of HY, while in the other years, those of CFR and DMR were always higher compared to that of HY. The average rice yields of CFR and DMR in year 9 were 2.0% and 2.9% higher compared to that of HY, respectively. This is consistent with the research of Wu et al. [31].

### 4.2. Straw and Total Biomass Production

Aboveground total biomass is a direct reflection of crop growth [32]. This study showed that in the early stage of the experiment, the straw yield of organic fertilizer partially replacing chemical fertilizer was higher than that of chemical fertilizer treatment alone. However, the straw yield of the latter exceeded that of the former, and the total biomass of the latter was lower than that of the former. This shows that partly replacing chemical fertilizer with an organic fertilizer in long-term rice planting can optimize the material distribution and lead to the transfer of more nutrients to the harvestable (grain), thus increasing the rice harvest index while improving the total biomass [33]. This is mainly due to the slow supply of nutrients in the early stage and the low accumulation of the aboveground biomass of rice. With the increase in the number of test years, organic fertilizer improves the soil environment, promotes the absorption of nutrients by rice, and thus increases the total biomass [34]. In addition, after partly replacing chemical fertilizer with organic fertilizer, the nutrient supply becomes more consistent with the absorption law of rice, thus promoting more nutrient transfer to the rice for harvest [35].

### 4.3. Nutrient Concentrations and Nutrient Uptake in Plants

The crop nitrogen concentration is mainly affected by the nitrogen application rate and nitrogen fertilizer form [29]. The forms of nitrogen used in organic fertilizer are diverse, and nitrogen can be better absorbed by, and transported in, crops after combined application with chemical fertilizer [36]. In this study, partly replacing chemical fertilizer with organic fertilizer could improve the nitrogen concentration of the rice grain. The nitrogen concentration of the DMR grain was significantly higher than that of HY and RF treated with chemical fertilizer alone. However, after partly replacing chemical fertilizer with organic fertilizer, the straw concentration was improved to some extent, but there was no significant difference compared to HY. Liao et al. [37] found that the concentration of nitrogen in rice grain and straw was increased after applying chemical urea in combination with milkvetch, which is consistent with the results of our study.

Fertilization can significantly increase the nitrogen uptake of rice [38]. Generally, a positive correlation exists between the rice yield and nutrient absorption. Xu M et al. [39] showed that the combined application of organic and inorganic fertilizers was beneficial to the nitrogen absorption of rice. In this study, the nitrogen uptake of rice for each treatment was consistent with the yield. The absorption of nitrogen induced by DMR was higher than that of CFR. Organic fertilizer contains slow-acting nitrogen fertilizer, the effect of which is equivalent to delayed nitrogen application and conducive to improving the rice’s absorption of nitrogen for its nutrition [40]. The partial substitution of chemical fertilizer with organic fertilizer mainly improves nitrogen absorption through an increased nitrogen concentration in plants and total aboveground biomass [41].

### 4.4. Fertilizer Use Efficiency of Rice and Nutrient Harvest Index

Nitrogen fertilizer use efficiency can directly reflect the rationality and effect of fertilization. A single application of chemical nitrogen fertilizer is not conducive to the balance of soil nutrients in farmland, and excessive application will lead to a decline in crop nitrogen fertilizer use efficiency [42].

Organic fertilizer replacement can effectively reduce the required level of fertilizer application, promote the absorption of nutrients by crops, and thus improve the fertilizer utilization efficiency [43]. In this study, compared with chemical fertilizer application alone, the combined application of organic–inorganic fertilizer variably improved the seasonal nitrogen recovery rate, agricultural utilization rate, and partial N productivity. This is mainly due to the fast release and easy loss of available N in chemical fertilizer, while the N in organic fertilizer is released slowly. This N release is better suited to crops’ nutrient demands, thus improving the nutrient utilization efficiency.

Research conducted by Miao et al. [44] showed that the long-term replacement of chemical fertilizer with organic fertilizer could improve the crop nitrogen harvest index. However, this study reached the opposite conclusion, mainly because the nitrogen uptake of grain under the chemical fertilizer treatment decreased. Meanwhile, the aboveground total nitrogen uptake also decreased due to the reduction in total biomass, which led to little change in the ratio between them.

### 4.5. Soil Chemical and Physical Properties

A scientifically and reasonably based fertilization system is one of the most important methods for preventing soil productivity degradation. Increased application of organic fertilizer is conducive to maintaining long-term and sustainable soil production [45]. Organic fertilizer can promote the formation of soil aggregates and improve soil physical structure, thereby increasing the nutrient and water retention capacity of soil and reducing the soil bulk density [46]. Wen et al. [47], by means of a 26-year positioning experiment, found that compared with chemical fertilizer application alone, the combined application of organic and inorganic fertilizers increased the soil organic matter and nutrient content and reduced the soil bulk density. Similar conclusions were obtained in our study. The effects of the combined application of organic and inorganic fertilizers on soil pH are not consistent. Tao et al. [48] showed that combining organic and inorganic fertilizers effectively improved the soil pH, which increased with the increasing proportion of organic fertilizer.

In contrast to Wen et al. [47], in this study, the pH of all fertilization treatments increased compared with the initial soil pH, but the organic replacement treatment resulted in a smaller increase. Differences in planting systems, geographical locations, soil properties, and experimental years might cause these different results.

## 5. Conclusions

The long-term replacement of chemical fertilizer with organic fertilizer slightly improves the rice yield (increased by 1.6–3.0% in this study), is more sustainable, and can increase the total biomass of the aboveground parts. In our study, the nitrogen uptake of the CFR and DMR treatments was also higher than that of the other treatments. The nitrogen recovery efficiency in the current season could be increased by 16.37–22.89% after 9 years of testing, and the soil total nitrogen contents of CFR and DMR increased by 0.23–0.85 g·kg^−1^ compared to the other treatments. The available P and K contents of DMR increased by 30.17 mg·kg^−1^ and 22.02 mg·kg^−1^ compared with HY, respectively. The soil bulk density was reduced by 0.08 g·cm^−3^. Generally, the effects of the dairy manure replacement were better compared to those of cake fertilizer. This is an important method that can be used to fertilize the soil and ensure sustainable soil utilization in the rice-growing area of the Yangtze River Basin, as well as a long-term partial replacement for chemical nitrogen fertilizer.

## Figures and Tables

**Table 1:** Effect of different fertilization treatments on yield components of rice.

Year	Treatment	PT/(10^4^·hm^−2^)	SP/(Panicle^−1^)	PFG/(%)	TGW/(g)
2012	CK	169.5b	178.9b	73.4a	27.5b
	HY	196.5a	224.1a	74.5a	27.9ab
	RF	178.8ab	209.5a	73.7a	27.5b
	CFR	187.0a	210.5a	78.5a	28.3a
	DMR	189.0a	213.7a	75.3a	28.3a
2013	CK	156.7b	182.9b	70.0a	27.0b
	HY	195.0a	240.1a	80.0a	30.6a
	RF	180.0ab	199.9b	79.4a	29.8a
	CFR	185.0ab	238.3a	76.7a	30.4a
	DMR	213.3a	213.3ab	78.9a	28.8ab
2014	CK	142.5c	184.3b	76.6a	27.1b
	HY	207.1a	219.9a	78.7a	29.0a
	RF	178.3b	195.2ab	77.7a	28.9a
	CFR	200.0ab	213.1ab	77.2a	29.2a
	DMR	199.2ab	212.6ab	78.3a	29.2a
2015	CK	138.8c	175.8b	74.0b	23.7b
	HY	201.1a	213.3a	78.8a	23.8b
	RF	171.8b	203.9a	80.8a	24.2ab
	CFR	205.2a	207.6a	79.6a	24.5a
	DMR	213.1a	212.4a	79.9a	24.3ab
2016	CK	136.5c	185.4b	78.3b	23.5a
	HY	197.7a	211.9a	81.8ab	24.0a
	RF	170.1b	204.9a	82.9ab	23.8a
	CFR	205.2a	217.4a	84.8a	24.1a
	DMR	208.6a	216.1a	86.0a	24.2a
2017	CK	154.7b	184.0b	76.7b	23.6b
	HY	208.3a	212.5a	82.2ab	24.2ab
	RF	196.4a	207.8a	83.8a	24.1ab
	CFR	212.3a	217.3a	84.4a	24.3a
	DMR	213.4a	220.1a	86.5a	24.3a
2018	CK	205.0b	196.4b	76.6a	21.9a
	HY	277.5ab	212.3a	79.8a	22.4a
	RF	247.5ab	201.9a	79.8a	22.4a
	CFR	282.5a	217.5a	76.1a	22.5a
	DMR	272.5ab	217.4a	81.4a	22.1a
2019	CK	181.8c	187.0b	76.5a	23.5c
	HY	253.5a	210.2ab	80.0a	25.4ab
	RF	190.5c	208.0ab	81.0a	25.6ab
	CFR	256.5a	212.1ab	79.6a	26.1a
	DMR	214.5b	217.2a	81.9a	24.2bc
2020	CK	184.5d	183.6b	77.1a	22.6b
	HY	259.5a	210.1a	80.1a	25.6a
	RF	205.5c	203.8a	80.9a	25.7a
	CFR	241.5b	213.2a	80.5a	25.9a
	DMR	243.0b	214.3a	81.8a	25.4a
		*F* value			
(Year) Y		31.98 **	1.096	7.456 **	159.858 **
(Treatment) T		55.07 **	32.719 **	9.785 **	19.579 **
Y × T		1.25	0.810	0.648	1.940

Note: Different lowercase letters in the same column show significant differences among treatments (*p* < 0.05) in the same year; (** *p* < 0.001).

**Table 2:** Effects of different fertilization treatments on rice yield and yield stability, t·hm^−2^.

Treatment	Year									Average Yields	Accumulative Yields	CV	SYI
	2012	2013	2014	2015	2016	2017	2018	2019	2020				
CK	7.4b	6.4c	5.5b	5.8c	6.9d	6.5c	6.9b	6.5b	6.4b	6.5c	58.2c	0.10a	0.773b
HY	10.2a	10.6a	10.3a	10.0a	9.9b	10.0ab	9.2a	11.2a	10.5a	10.2a	92.0a	0.07c	0.848a
RF	9.4a	9.8b	9.8a	8.4b	8.8c	9.2b	9.2a	10.6a	10.0a	9.5b	85.2b	0.08b	0.792b
CFR	9.8a	10.2ab	10.0a	10.6a	10.5ab	10.6a	10.2a	11.3a	10.4a	10.4a	93.5a	0.05d	0.867a
DMR	9.6a	10.5a	10.0a	10.8a	10.9a	10.9a	10.0a	11.5a	10.6a	10.5a	94.8a	0.07c	0.854a
					*F* value								
Year (Y)						7.647 **							
Treatment (T)						324.066 **							
Y × T						3.108 **							

Note: Different lowercase numbers indicate significant differences between treatments; *p* < 0.05. ** *p* < 0.001.

**Table 3:** Effects of different fertilization treatments on rice straw yield, total biomass, and harvest index.

Year	Treatment	Straw Yield (t·hm^−2^)	Total Biomass (t·hm^−2^)	Harvest Index
2012	CK	7.69b	15.09c	0.49b
	HY	9.30a	19.47a	0.52a
	RF	8.44ab	17.84b	0.53a
	CFR	9.23a	19.01ab	0.51b
	DMR	8.69a	18.32ab	0.53a
2013	CK	6.58b	12.97b	0.49b
	HY	9.81a	20.38a	0.52a
	RF	9.40a	19.20a	0.51ab
	CFR	9.86a	20.10a	0.51ab
	DMR	9.84a	20.35a	0.52ab
2014	CK	5.99b	11.46b	0.48b
	HY	9.11a	19.43a	0.53a
	RF	9.98a	19.74a	0.49b
	CFR	9.18a	19.18a	0.52a
	DMR	9.39a	19.40a	0.52a
2015	CK	6.02c	11.80c	0.49b
	HY	8.63ab	18.64a	0.54a
	RF	7.54b	15.92b	0.53a
	CFR	9.57a	20.18a	0.53a
	DMR	9.22a	19.98a	0.54a
2016	CK	7.08b	13.93c	0.49b
	HY	9.77a	19.66a	0.50b
	RF	7.59b	16.37b	0.54a
	CFR	8.93a	19.40a	0.54a
	DMR	9.13a	20.00a	0.54a
2017	CK	6.83b	13.29c	0.49c
	HY	10.03a	20.01ab	0.50bc
	RF	8.44a	17.61b	0.52ab
	CFR	9.82a	20.40a	0.52ab
	DMR	9.15a	20.01ab	0.54a
2018	CK	7.05b	13.99b	0.50c
	HY	8.66a	17.90a	0.52bc
	RF	8.39a	17.63a	0.52ab
	CFR	8.88a	19.07a	0.53a
	DMR	8.41a	18.44a	0.54a
2019	CK	6.54c	13.01b	0.50b
	HY	11.16a	22.47a	0.50b
	RF	10.27ab	20.92a	0.51b
	CFR	9.71b	20.87a	0.54a
	DMR	9.72b	21.24a	0.54a
2020	CK	7.79b	14.23b	0.45b
	HY	10.96a	21.43a	0.49ab
	RF	10.03a	20.01a	0.50 ab
	CFR	9.97a	20.42a	0.51a
	DMR	9.92a	20.51a	0.52a

Note: Different lowercase letters in the same column show significant differences among treatments (*p* < 0.05) in the same year.

**Table 4:** Effects of different fertilization treatments on nitrogen utilization efficiency and nitrogen harvest index (2020).

Treatment	RE_N_ (%)	AE_N_ (kg/kg)	PFP_N_ (kg/kg)	Nitrogen Harvest Index (%)
CK	-	-	-	63.71a
HY	38.27c	14.95a	38.80b	62.70a
RF	49.52b	16.88a	47.55a	60.23a
CFR	54.64ab	19.09a	49.75a	61.13a
DMR	61.16a	19.72a	50.39a	63.89a

Note: Different lowercase letters in the same column show significant differences among treatments (*p* < 0.05).

**Table 5:** Effects of different fertilization treatments on soil properties.

Treatment	pH	Organic Matter/(g·kg^−1^)	Total N/(g·kg^−1^)	Available P/(mg·kg^−1^)	Available K/(mg·kg^−1^)	Bulk Density/(g·kg^−3^)
CK	7.13a	23.85d	1.41b	8.75c	110.83d	1.26a
HY	7.00a	31.53c	1.86ab	29.84b	209.22b	1.16bc
RF	7.01a	30.28c	1.79ab	12.48bc	132.91c	1.20ab
CFR	6.97a	35.48b	2.09a	18.17bc	194.92b	1.13bc
DMR	6.96a	39.43a	2.26a	60.01a	231.24a	1.08c

Note: Different lowercase letters in the same column show significant differences among treatments (*p* < 0.05).

## Data Availability

The authors confirm that the data supporting the findings of this study are available within the article.
